# Multiple genotypes within aecial clusters in *Puccinia graminis* and *Puccinia coronata*: improved understanding of the biology of cereal rust fungi

**DOI:** 10.1186/s40694-017-0032-3

**Published:** 2017-05-03

**Authors:** Anna Berlin, Berit Samils, Björn Andersson

**Affiliations:** Department of Forest Mycology and Plant Pathology, Swedish University of Agricultural Sciences, Box 7026, 750 07 Uppsala, Sweden

**Keywords:** Stem rust, Crown rust, Cereal rusts, Life cycle

## Abstract

**Background:**

Cereal rust fungi (*Puccinia* spp.) are among the most economically important plant pathogens. These fungi have a complex life cycle, including five spore stages and two hosts. They infect one grass host on which they reproduce clonally and cause the cereal rust diseases, while the alternate host is required for sexual reproduction. Although previous studies clearly demonstrate the importance of the alternate host in creating genetic diversity in cereal rust fungi, little is known about the amount of novel genotypes created in each successful completion of a sexual reproduction event.

**Results:**

In this study, single sequence repeat markers were used to study the genotypic diversity within aecial clusters by genotyping individual aecial cups. Two common cereal rusts, *Puccinia graminis* causing stem rust and *Puccinia coronata* the causal agent of crown rust were investigated. We showed that under natural conditions, a single aecial cluster usually include several genotypes, either because a single pycnial cluster is fertilized by several different pycniospores, or because aecia within the cluster are derived from more than one fertilized adjoining pycnial cluster, or a combination of both.

**Conclusion:**

Our results imply that although sexual events in cereal rust fungi in most regions of the world are relatively rare, the events that occur may still significantly contribute to the genetic variation within the pathogen populations.

**Electronic supplementary material:**

The online version of this article (doi:10.1186/s40694-017-0032-3) contains supplementary material, which is available to authorized users.

## Background

Cereal rust fungi (*Puccinia* spp.) are among the most studied plant disease-causing agents, as they affect cereals and grasses in all parts of the world, potentially causing devastating yield losses. Some of the most important cereal rust diseases are stem rust (caused by *P. graminis*), stripe rust (*P. striiformis)*, leaf rust on wheat (*P. triticina*) leaf rust on rye (*P. recondita*), barley leaf rust (*P. hordei*) and crown rust on oats (*P. coronata*) [1, 2]. Cereal rust species can be subdivided based on their host specificity [3], and all cereal rusts may infect a wide variety of wild grass species [4]. The different specializations are referred to as *formae speciales* (f. sp.), i.e. the wheat-infecting *P. graminis* is referred to as *P. graminis* f. sp. *tritici* and the oat-infecting type as *P. graminis* f.sp. *avenae* [3].

Cereal rust fungi are obligate biotrophs that have macrocyclic and heteroecious life cycles, including five spore stages and two hosts. The gramineous hosts enable efficient clonal reproduction while the alternate hosts are necessary for sexual reproduction, which constitutes an important source of genetic diversity [5]. The spore stage causing disease on cereals and other grasses is the uredinial stage, where clonally propagated dikaryotic urediniospores re-infect the gramineous hosts in several infection cycles. The urediniospores are thick-walled and withstand solar irradiation [6], which enable rusts to potentially spread with winds over large distances [7]. As the gramineous host matures, the fungus initate teliospore production. The teliospores of *P. graminis* appear in the same pustules (or sori) as the urediniospores, while teliospores of *P. coronata* create a characteristic blackish ring around the pustule of urediniospores [8]. In temperate climates, the teliospores are the overwintering spores. *Puccinia graminis* and *P. coronata* undergo karyogamy in autumn, whereupon meiosis starts and is completed in spring when the teliospores germinate and haploid basidiospores develop [9, 10]. The basidiospores can only disperse in the range of hundreds of meters [11] before infecting young tissue of the alternate hosts, where pycnia and pycniospores (syn. spermogonia and spermatia) develop. One infection originating from a single basidiospore usually gives rise to multiple pycnia in a tight group (pycnial cluster) on the upper side of the host leaf [12]. The pycniospores are produced in a sugary nectar that attracts insects that act as vectors and transfer the spores between pycnia [10, 13]. Pycniospores can also disperse by splashing raindrops. When a pycniospore from one pycnial cluster successfully fertilizes the receptive hyphae of a pycnial cluster of the opposite mating type, a dikaryotic mycelium is formed which grows towards the lower side of the leaf where an aecium develops, and dikaryotic aeciospores are formed. The aecium has a cup-like form and usually appears close together with other aecia in an aecial cluster (or cup cluster), opposite the pycnial cluster [13]. It has been suggested that spores within an aecium are genetically identical [8, 14]. The aeciospores are released from the aecia and infect the gramineous hosts, where new uredinia and urediniospores are produced.

The role of the alternate hosts of rust fungi on cereals has regained attention after the emergence of the virulent *P. graminis* race Ug99 [15] and the discovery of barberry as the aecial host of the stripe rust fungus *P. striiformis* [16]. The life cycle of *P. graminis* has been known for centuries [17], and both *P. striiformis* and *P. graminis* have the shrub barberry (*Berberis* spp.) and Mahonia (*Mahonia* spp.) as their alternate hosts [16, 17]. The alternate hosts for *P. coronata*, the fungus causing crown rust on oats and other grasses, are *Rhamnus* spp. and *Frangula alnus*. However, the literature reports that the *forma specialis* infecting oats only produce aecia on *Rhamnus cathartica* [4, 8].

Jin et al. [18] showed that the presence of the alternate hosts *Berberis vulgaris*, *Mahonia repens* and *M. aquifolium* in North Western United States of America maintained the diversity within the *P. graminis* population, whereas it has declined to a single clonal linage on the west side of the Rocky Mountains where barberry has been successfully eradicated. Other studies also show that the genetic diversity within the pathogen populations on the gramineous hosts are high in regions where the growing seasons are clearly separated and the alternate host are needed for the rusts survival [19–21]. In areas where the fungus can survive year-round in the clonal, uredinial, stage on its gramineous host, the genetic diversity originating from sexual reproduction is suggested to be limited. One example of this is the Ug99 race lineage of *P. graminis.* This race was first detected in Uganda in 1999 [15] and fifteen years later it dominated the *P. graminis* population in East Africa [22].

Traditionally, the studies of variation within cereal rust fungi have been based on race analysis where virulence against known resistance genes is tested. Many rust resistance genes have been identified and characterized [23]. The race identification is performed by inoculating urediniospore isolates on a set of differential host lines, each carrying different resistance genes. Specific virulence phenotypes (races or pathotypes) are defined by the phenotypic reactions on the differential host lines, giving a specific resistance pattern for each fungal isolate [24, 25]. Earlier studies, using race analysis of uredinial offspring, show that phenotypically different individuals can be produced from a single aecial cluster [26, 27]. Although virulence phenotyping is useful for its purpose, it will not identify overall genetic variation or separate among individual genotypes efficiently. By applying molecular methods and DNA-based markers, genetic relationships among fungal isolates can be determined based on neutral markers and with higher resolution [28].

In our study, we aimed to determine the genotypic diversity after a single completion of the sexual part of the life cycle of cereal rust fungi under natural conditions. Aecial clusters from natural infections of *P. graminis,* collected on *B. vulgaris,* and *P. coronata*, collected on *R. cathartica* and *F. aluns,* were analyzed. The genotypic diversity among cups within single aecial clusters on the alternate hosts of these fungi was investigated using single sequence repeat (SSR) markers.

## Results

In total, the eight SSR markers used to analyze the aecial collections of *P. graminis* identified 46 alleles, and the ten SSR markers used in the aecial collections of *P. coronata* identified 40 alleles (Table 1). For *P. coronata*, the allele distribution showed a clear population differentiation between the two aecial hosts *F. alnus* and *R. cathartica*. Only one of the identified alleles (locus PcaSSR B02, allele 167) was shared between samples collected from the two hosts. One SSR marker (PcaSSR A66) completely failed to amplify in the samples collected from *F. alnus,* and one (PcaSSR B25) only amplified two out of 18 samples (Additional file 1: Table S1).

**Table 1:** Allele sizes of SSR loci for *Puccinia coronata* and *Puccinia graminis*

*Puccinia coronata*	*Rhamnus cathartica*	*Frangula alnus*	*Berberis vulgaris*
PcaSSRA59^a^	137, 140, 143	134	
PcaSSRA66^a^	175, 185, 187		
PcaSSRA67^a^	189, 191	172	
PcaSSRA73^a^	150	156, 160	
PcaSSRB02^a^	159, 161, 164, 167*	167*, 172	
PcaSSRB09^a^	142, 151	125	
PcaSSRB25^a^	197, 201, 203, 205		
PcaSSRB33^a^	182, 186, 188, 196	166	
PcaSSRC52^a^	192, 200, 202	190	
PcaSSRC76^a^	143	137, 139, 141, 152	
***Puccinia graminis***			
Pgestssr021^b^			231, 237, 240, 243, 246, 249, 252
PgtSSR21^c^			167, 169, 171, 173, 175
Pgestssr024^b^			121, 130, 133, 156, 159
Pgestssr109^d^			159, 162, 168, 171, 174
Pgestssr255^b^			228, 231, 234, 237, 240, 243, 246
Pgestssr279^d^			169, 175, 178, 181, 184
Pgestssr368^d^			229, 232, 235, 238, 241, 245, 248
PgtCAA53^e^			176, 185, 202, 211, 214

* Allele shared between hosts

Source of SSR markers: ^a^ Dambroski et al. [29], ^b^ Berlin et al. [20], ^c^ Szabo [30], ^d^ Zhong et al. [31], ^e^ Jin et al. [32]

In total 22 aecial clusters were analyzed; 12 representing *P. coronata,* where 7 were excised from *R. cathartica* and 5 from *F. alnus*, and 10 representing *P. graminis* which were all excised from *B. vulgaris* (Table 2, Additional file 1: Table S1 and Additional file 2: Table S2). The number of aecial cups analyzed for each cluster reflects the size of the particular cluster, and ranged between 2 and 21 cups genotyped (N), with an overall average of 8.4 cups genotyped per cluster.

**Table 2:** Number of aecial cups genotyped (N) and number of identified multilocus genotypes (MLGs) within each aecial cluster of *Puccinia coronata* and *Puccinia graminis* respectively, collected on *Rhamnus cathartica*, *Frangula alnus* and *Berberis vulgaris*

	Host	N	MLG
*P. coronata*			
1604	*Rhamnus cathartica*	5	4
1605	*Rhamnus cathartica*	21	10
1606	*Rhamnus cathartica*	6	6
1607	*Rhamnus cathartica*	6	4
1608	*Rhamnus cathartica*	4	3
1609 A–G	*Rhamnus cathartica*	7	6
1609 H–J	*Rhamnus cathartica*	3	2
1611 A–C	*Fragula aluns*	3	1
1611 D–F	*Fragula aluns*	3	2
1611 G–M	*Fragula aluns*	7	6
1611 N–O	*Fragula aluns*	2	2
1611 P–R	*Fragula aluns*	3	3
Total		70	49
*P. graminis*			
1601	*Berberis vulgaris*	13	4
1603	*Berberis vulgaris*	16	9
1612 A–F	*Berberis vulgaris*	6	3
1612 G–I	*Berberis vulgaris*	3	2
1612 J–O	*Berberis vulgaris*	6	2
1613	*Berberis vulgaris*	14	7
1614	*Berberis vulgaris*	17	5
1615	*Berberis vulgaris*	15	6
1616	*Berberis vulgaris*	7	4
1617	*Berberis vulgaris*	18	8
Total		115	49

**Fig. 1:**
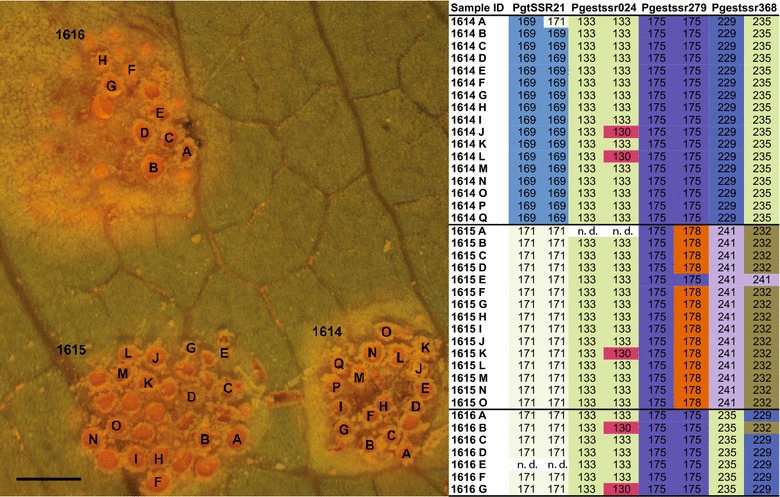
*Puccinia graminis* aecial clusters and SSR allele sizes of selected markers. *Bar* 1 mm

**Fig. 2:**
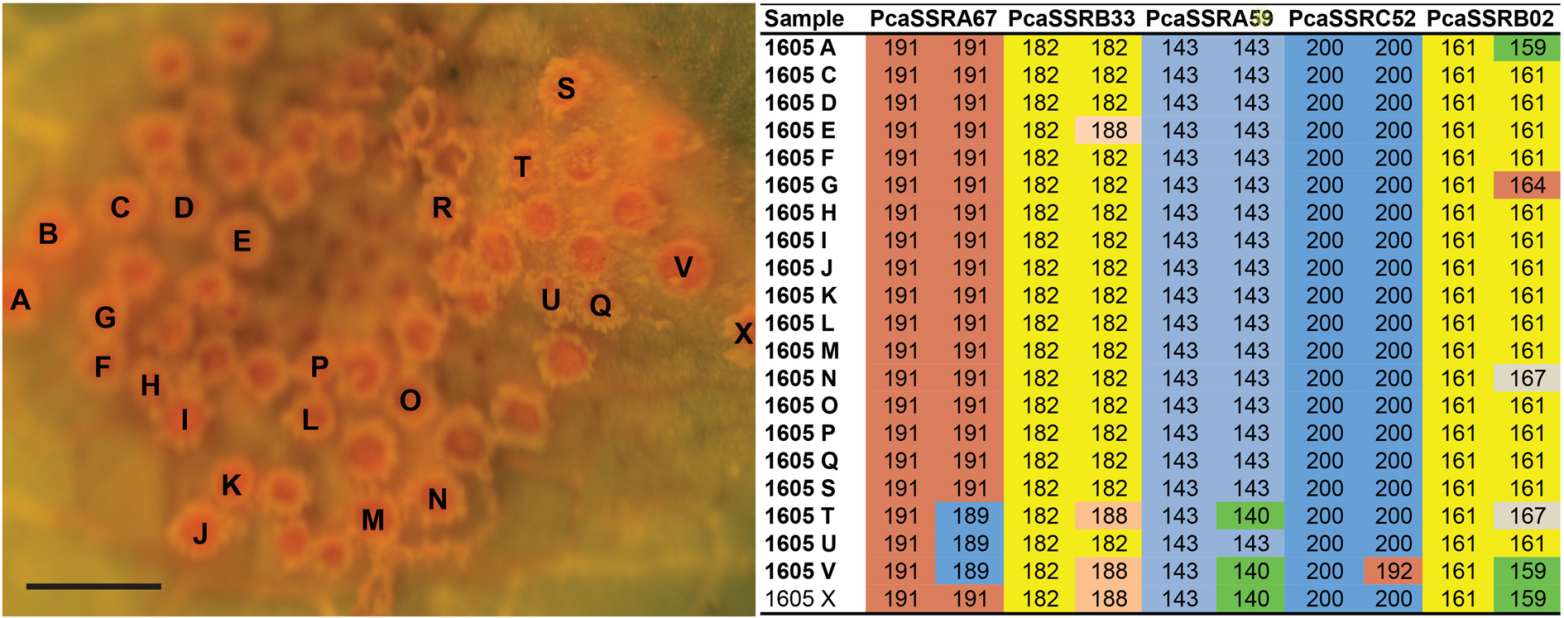
*Puccinia coronata* aecial cluster and SSR allele sizes of selected markers. *Bar* 1 mm

The multilocus genotype (MLG) for each individual aecial cup (aecium) was determined by the combination of the alleles from all individual SSR markers for that particular sample. Multiple genotypes were detected in all but one aecial cluster, with an average of 4.1 MLGs for *P. coronata* and 4.9 MLGs per cluster for *P. graminis* (Table 2). It should be noted that in particular for *P. coronata*, the high number of non-detected alleles for samples collected from *F. alnus* could lead to an underestimation of the number of MLGs. Within each aecial cluster, in most cases, one of the two alleles for each SSR locus is invariant among aecial cups whereas the other allele may differ (Figs. 1, 2). A few exceptions to this pattern were detected in three of the 10 clusters in *P. graminis*, where one or a few aecial cups did not share the common allele with other cups in the same cluster (cluster cups 1603 D-H; 1613 A-D; 1617 A, Additional file 2: Table S2).

## Discussion

The present study aimed at determining the fine-scale genotypic diversity in the aecial stage of cereal rust fungi. Our results showed that under natural conditions, different genotypes are usually produced within each aecial cluster on the alternate host of both *P. graminis* and *P. coronata*. This implicates that a single mating event will contribute substantially to the genetic variation in a cereal rust fungal population. Multiple genotypes within aecial clusters have previously been indicated by virulence phenotyping [26, 27]. After artificial fertilization of one pycnial cluster, aeciospores from a single aecial cup (or aecium) produced one virulence phenotype, while other cups within the same aecial cluster produced offspring with a different phenotype. Here we used molecular markers to demonstrate that multiple genotypes within aecial clusters are common in nature, and as many as ten different multilocus genotypes were detected within a single cluster.

Multiple genotypes within an aecial cluster suggest multiple fertilization events within a pycnial cluster. We showed that the aecial cups within a cluster usually shared one allele for each SSR loci, while the second allele in many cases differed between the cups. This pattern indicates that one pycnial cluster (consisting of pycnia with identical genotypes) are fertilized by genotypically different pycniospores, i.e. coming from different pycnial clusters. The shared allele would thus originate from the resident pycnial cluster, and the second allele would originate from the fertilizing pycniospores. This pattern was observed for both *P. graminis* and *P. coronata*, indicating that it may be consistent across different cereal rust species. A similar allelic pattern has been observed in the blister rust fungus of Scots pine, *Cronatium flaccidum,* where one allele was shared within aecial lesions, indicating multiple mating’s between the spermogonium (syn. pycnium) and fertilizing spermatia (syn. pycniospores) [33]. In nature, multiple fertilizations are supported by insects who are attracted by the pycnispore-containing nectar in the pycnia and travel from leaf to leaf and transfer pycniospores among pycnia. It is also possible that closely located pycnial clusters may contribute to the same aecial cluster, and in such case both alleles of the SSR loci might differ between some aecial cups. Such a pattern was not common in the present study, but was detected in a few cases for *P. graminis* (Additional file 2: Table S2).

As a result of meiosis taking place in the teliospore, the offspring produced from a selfing of a clonal linage of a rust fungus will be genetically diverse. This was demonstrated by two recent studies [14, 34]. For example, Tian et al. [34] selfed a *P. striiformis* isolate, resulting in 118 viable offspring’s determined as 82 multilocus genotypes and 24 virulence phenotypes. When diversity is described by virulence phenotyping, the true genotypic diversity will be underestimated, since only variation caused by a limited number of virulence genes is assessed, and additionally, virulence genes are usually recessive und can thus be hidden in the avirulent phenotypes. The study by Tian et al. [34] clearly shows this discrepancy and it also illustrates the level of overall genetic variation originating from sexual recombination within a single clonal linage. These results, in combination with our findings, imply that there will be large amounts of genetic and genotypic variation in the aeciospore inoculum reaching and infecting the grass hosts.

Our results on host specific allele sizes of SSR loci confirm the usefulness of SSR markers to distinguish between *formae speciales* of *P. coronata*, as they agree with the previously described DNA sequence based genetic differentiation of the two *formae speciales* [35] and their ability to only infect specific alternate hosts [4].

A practical implication of our results for population genetic studies as well as virulence phenotyping is the scale of sampling required and interpretations of virulence tests. Algorithms in population genetic analyses are often based on single isolates (i.e. single individuals) and thus sampling in the aecial stage of cereal rust fungi should be done on individual aecial cups, rather than entire aecial clusters. If sampling and analyses are done on genotypic mixtures it may give confusing and erroneous results. The same is also applicable for virulence phenotyping. If mixtures of aeciospore genotypes are tested, the results could imply complex virulence races where in reality simple virulence races were mixed together. Additionally, there is some inconsistency in terminology of aecial morphology, which could lead to misinterpretations. According to many textbooks and other literature [14, 36, 37], a single cup in an aecial cluster of *P. graminis* represents an aecium. However in some studies, the whole aecial cluster (with several aecial cups) is denoted as an aecium [38, 39]. In other studies, it may be unclear whether an aecium refers to a single aecial cup or a cup cluster.

## Conclusions

The genetic diversity of the uredinial, clonal stage of both *P. graminis* and *P. coronata* on the cereal hosts, has been extensively studied in several areas of the world. In the present study, we emphasise the importance of the aecial stage on the alternate host in creating genotypic diversity, where one successful completion of the sexual cycle in natural condition may result in aecial clusters containing several novel genotypes. This means that genetic diversity is introduced to the cereal rust fungal populations more efficiently than previously acknowledged. The main conclusion of our result is that although sexual events of cereal rust fungi in most regions of the world are relatively rare, the events that do occur may still contribute significantly to the genetic variation within the pathogen populations.

## Methods

Collections of natural infected leaves showing clear symptoms of cluster cup rust were performed at two locations (Hågadalen N59°49′16″ E17°36′4″ and Skerike N59°37′40″ E16°30′13″) in late June. Both locations were located approximately 500 m from the closest cereal fields. Samples of *Berberis vulgaris* and *F. alunus* leaves were collected in Hågadalen and *R. cathartica* leaves in Skerike. The leaf samples were put in a plant press before returning to the laboratory.

The aecia were photographed and single aecial cups were carefully cut to enable genotyping of the individual cups within an aecial cluster. Sample identities were indicated on the photo for later correlation between sample position within an aecial cluster and genotype. Aecia on *B. vulgaris* and *R. cathartica* were commonly found and were excised from both the same and different leaves. However, aecial clusters on *F. alnus* were scarce and all were excised from the same leaf. For *P. coronata*, 7 aecial cluster were sampled from *R. cathartica* and 5 from *F. alnus*, and 3–21 and 2–7 individual cups per cluster, respectively, were successfully genotyped. For *P. graminis*, 10 aecial clusters were sampled, and 3–18 aecial cups were successfully genotyped. An average of 7 cups per cluster was sampled, the differences in number of samples largely reflect the actual number of aecial cups within the sampled cluster.

For each of the sampled aecial cups, DNA was extracted using the OmniPrep Kit (G-Biosciences) for fungal tissues, with the adjustment of the amounts of reagents to half to adjust for small sample sizes. Eight single sequence repeat (SSR) markers developed for *P. graminis* [20, 30–32] and 10 SSR markers developed for *P. coronata* [29] were applied on the different fungal samples to study the genetic diversity within the aecial clusters (Table 1). The PCR reaction was performed in a 14 μl reaction with the final concentration of 14 ng DNA, 0.4 μM of the each forward and reverse primers respectively, 3.5 mM MgCl_2_, 0.02 mM of each of the dNTP, 0.05 U/μl of DreamTaq DNA polymerase and Dream Taq buffer according to manufacturer’s recommendation (Thermo Fischer Scientific). PCR conditions were initial denaturation at 94 °C, followed by 35 cycles of denaturation at 94 °C for 30 s, annealing at 55 °C for 30 s and extension at 72 °C for 30 s, and a final extension of 72 °C for 10 min. The length of the amplicons was determined using ABI 3730xl DNA Analyzer (SciLifeLab, Uppsala, Sweden) and was scored using the software GeneMarker (Softgenetics). To calculate the number of genotypes within each aecial cluster, the R add-in software PoppR was used [40].

## Additional files

**Additional file 1.** SSR allele sizes of all aecia within aecial clusters for *Puccinia coronata*, sampled from the hosts *Rhamnus cathartica* and *Frangula alnus*.

**Additional file 2.** SSR allele sizes of all aecia within aecial clusters for *Puccinia graminis*, sampled from the host *Berberis vulgaris*.
